# Postexercise substrate oxidation is environmental temperature dependent before and after short‐term exercise training in obese women

**DOI:** 10.1002/ejsc.12032

**Published:** 2024-01-30

**Authors:** Onanong Kulaputana, Parimon Kaewpaluk, Sompol Sanguanrungsirikul

**Affiliations:** ^1^ Sports Medicine Program Faculty of Medicine Chulalongkorn University Bangkok Thailand; ^2^ Sports and Exercise Medicine Research Laboratory Department of Physiology Faculty of Medicine Chulalongkorn University Bangkok Thailand; ^3^ King Chulalongkorn Memorial Hospital Bangkok Thailand

**Keywords:** ambient heat exposure, energy expenditure, obesity, recovery metabolism, respiratory exchange ratio, weight management

## Abstract

Substrate oxidation can be altered by both environmental temperature and exercise training. It is unclear whether environmental temperatures before and after short‐term exercise training influence substrate oxidation rates and energy expenditure (EE) during postexercise recovery. The purpose of this paper is to study the effects of hot and thermoneutral environments on substrate oxidation and EE during postexercise recovery before and after a 1‐month exercise training in obese women. Sixteen overweight or obese women underwent a 1‐month exercise training. Before and after training, each participant completed metabolic testing during postexercise recovery at either hot (31°C–32°C) or thermoneutral (22°C–23°C) environments in a randomized crossover fashion with a washout period of 2–4 days between the two tests. The substrate oxidation and EE determined by indirect calorimetry during the 60‐min postexercise recovery of the hot and thermoneutral environments were compared. Following exercise training, fat oxidation during recovery was significantly greater at thermoneutral than at hot environments (thermoneutral, 56.0 ± 24.6 mg/kg/h vs. hot, 39.7 ± 27.5 mg/kg/h; *p* < 0.001). Conversely, carbohydrate oxidation during the recovery was significantly greater at hot than at thermoneutral environments, and the total EE at both temperatures did not significantly differ (hot, 70.5 ± 19.6 kcal/h vs. thermoneutral, 71.3 ± 13.7 kcal/h; *p* = 0.846). The results were the same as those before exercise training. After an acute bout of exercise, recovery in a thermoneutral environment increases fat oxidation; however, environmental temperatures produce no effect on the total EE. The same results were obtained before and after exercise training, suggesting that energy and substrate metabolism during postexercise recovery are more influenced by the environmental temperature than exercise training.

## Key Points

- Substrate oxidation during postexercise recovery can be influenced by environmental temperature in obese women.
- Recovery in the thermoneutral environment results in greater fat oxidation than in the hot environment. EE is unaffected by the recovery environment.
- Exercise training does not alter the temperature‐related substrate oxidation or EE during recovery.

## 1 INTRODUCTION

Obesity has become a global epidemic and a significant public health problem given its association with many chronic diseases (World Health Organization, 2004). Together with altered energy intake, increased physical activity is necessary for successful weight management (Donnelly et al., 2009; Jakicic Chair et al., 2001). Physical exercise increases energy expenditure (EE) during and following the exercise bout (Borsheim & Bahr, 2003; Horton et al., 1998). Exercise intensity and duration affect fat oxidation and the total EE, with greater fat oxidation observed during the recovery period after exercising for a longer duration (Warren et al., 2009) and higher intensity (Phelain et al., 1997). The American College of Sports Medicine recommends that overweight and obese people engage, after appropriate progression, in moderate‐intensity aerobic exercise at a 40%–59% of heart rate reserve (HRR) lasting 30–60 min/day for 5 or more days a week or a total of 250–300 min/week (American College of sports medicine, 2016).

Substrate utilization and EE are also influenced by ambient temperatures. Heat stress increases carbohydrate oxidation both at rest and during exercise (Jentjens et al., 2002; Maunder et al., 2020; Pilch et al., 2010) Varying degrees of cold exposure, with (Gagnon et al., 2013) or without (Acosta et al., 2018; Haman et al., 2002; Tikuisis et al., 2000) exercise, shifts energy utilization to burning fat rather than carbohydrates. Most studies on the effects of environmental temperature on substrate metabolism during rest and exercise have been conducted with healthy individuals and athletes (Gagnon et al., 2013; Jentjens et al., 2002; Pilch et al., 2010; Tikuisis et al., 2000; Yaspelkis et al., 1993). However, the comparable information available for obese individuals is limited, especially during postexercise recovery. A previous study regarding substrate oxidation during postexercise recovery after moderate‐intensity exercise in obese women demonstrated that fat oxidation was greater when the participants recovered at thermoneutral (24°C–25°C) rather than hot (31°C–32°C) temperature ranges (Kulaputana et al., 2020). This finding suggested that ambient temperatures affect the metabolic pathways of substrate oxidation not only during rest and exercise but also during postexercise recovery.

A hot and humid environment impairs heat transfer from the body to the environment, which may be detrimental to the body's thermoregulatory response (Wendt et al., 2007). Hence, obese individuals who may be at risk of impaired thermoregulation should be careful while selecting the appropriate environmental conditions for exercising and postexercise recovery. Additionally, obese people are advised to exercise regularly, which may aid metabolic adaptation induced by chronic exercise training. Various exercise training programs lasting 4–10 weeks have been shown to augment fat oxidation both at rest (Jabbour et al., 2017; Lefai et al., 2017) and during exercise (Botero et al., 2014; Lanzi et al., 2015; Venables et al., 2008) in overweight and obese participants. This raises a question regarding whether the influence of environmental temperature on substrate oxidation and EE during postexercise recovery would be different before and after exercise training. This concern is of practical importance since hot outdoor temperatures in many regions worldwide compounded by global warming may increasingly impact human health. To address this question, we compared the effect of hot and thermoneutral environments on substrate oxidation and EE during postexercise recovery before and after a 1‐month exercise training in overweight and obese women.

## 2 MATERIALS AND METHODS

### 2.1 Study sample and design

Sixteen overweight (*n* = 3) or obese (*n* = 13) female participants (body mass index, mean [range], 33.4 [28.9–39.7] kg/m^2^; aged 33.5 [23–43]) were included in the study. Candidates with high blood pressure, diabetes, cardiac disease, and irregular menstrual cycles in the preceding 3 months or the ones using contraceptive hormones, diet pills, and drugs that affect body metabolism were excluded. Candidates whose body mass changed by >3 kg during the preceding 6 months were also excluded. Of the 20 women initially enrolled, 4 withdrew from the study owing to early menstruation (1 participant), delayed menstruation (1 participant), and the malfunction of the metabolic equipment that prevented on‐time testing (2 participants), leaving 16 participants in total.

**FIGURE 1:**
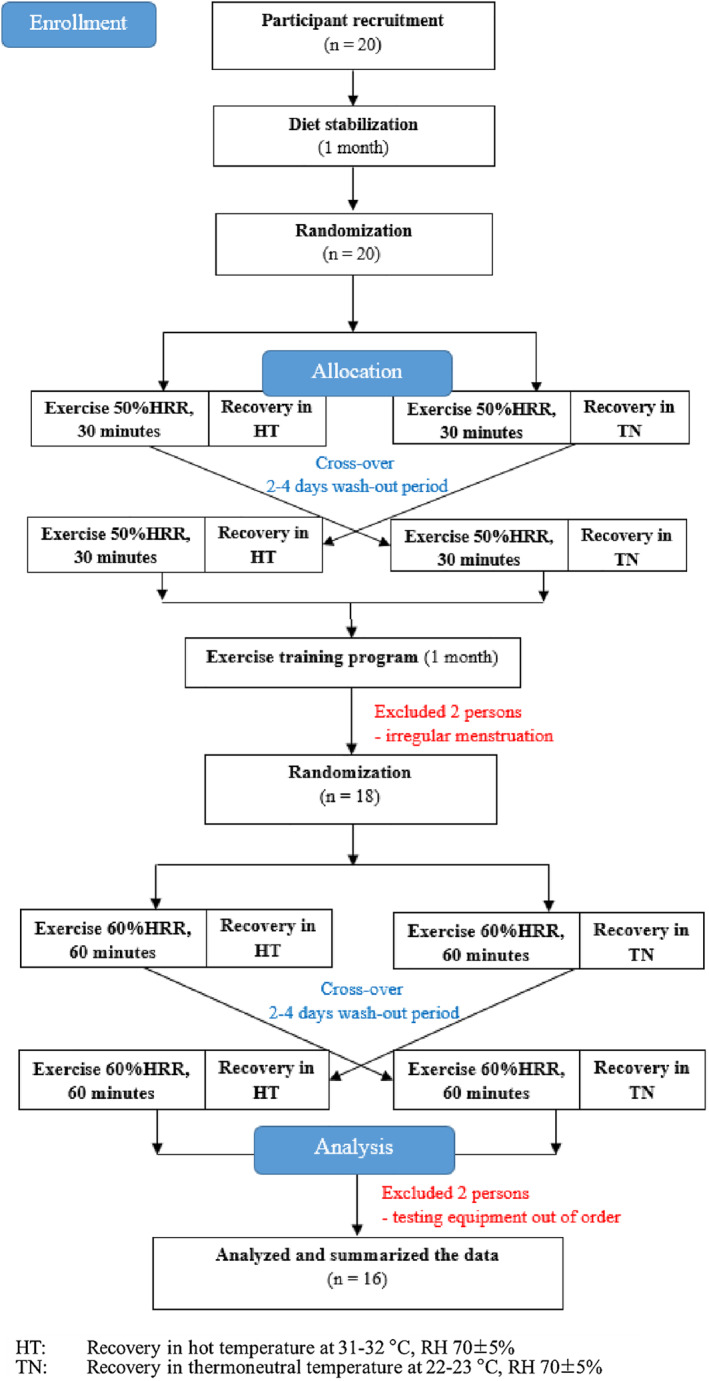
CONSORT and research flow diagram.

All the participants exercised in the same thermoneutral environment. Following the exercise, they entered a recovery phase in one of two randomly selected conditions, thermoneutral or hot, which was repeated after a 2–4 days washout period (randomized crossover trials). They underwent a second set of randomized crossover trials after a 1‐month exercise training program. The main outcome measures were substrate oxidation and EE during postexercise recovery. The CONSORT and research flow diagram of the experiment is shown in Figure 1.

### 2.2 Diet stabilization

Before exercise training, the participants attended a 3‐h class on healthy eating conducted by a nutritionist. They had to stabilize their diets by eating healthy for 1 month before the exercise training. Diet stabilization was implemented to minimize the influence of dietary variation on the study results owing to diet‐induced changes in the body mass and metabolism. The participants recorded their daily dietary intake for 3 days/week and continued with diet stabilization throughout the exercise training period, keeping diet records once weekly. The nutritionist evaluated the diet records every week using computer software (Inmucal version 3, Institute of Nutrition, Mahidol University), and gave individual feedback regarding food calories and nutrition to each participant.

### 2.3 Exercise training program

All the participants underwent the same supervised, progressive treadmill exercise. The intensity and duration of the training sessions started at a 50% HRR for 30 min and gradually progressed to a 60% HRR for 60 min by the second half of the third week, which was continued until the end of the training. The duration was increased by 5–10 min, and the intensity increased by 5% weekly. The speed and the grade of the treadmill were adjusted during the training sessions to achieve the prescribed target heart rate, which was predetermined based on pretraining resting HR. The participants performed four sessions per week during a full period of their individual menstrual cycle (∼1 month).

### 2.4 Indirect calorimetry

Indirect calorimetry was employed to estimate substrate oxidation and EE at rest, during exercise, and during postexercise recovery. On the day preceding each testing day, the participants were asked to consume the same type and amount of food that was consumed daily. The metabolic testing sessions were conducted in the morning after overnight fasting. Testing was scheduled in the early follicular phase (the first seven days) of the menstruation period to minimize the effect of ovarian hormones on substrate metabolism (Oosthuyse et al., 2010; D'Eon et al., 2002).

Expired gases were collected using a breath‐by‐breath portable gas analysis system (Jaeger, Oxycon mobile, Germany). Metabolic equipment was calibrated before each testing. Gas samples for metabolic measurement were averaged over the last 15 min of the total 30 min of supine duration for the resting condition, over the whole period of the exercise session at thermoneutral environments, and over 1 h during recovery in either thermoneutral or hot environments. The metabolic rates were calculated according to the stoichiometric formula of Peronnet and Massicotte (Péronnet et al., 1991), formalized by Equations ((1), (2), (3))–((1), (2), (3)) below.

(1) $$\text{Fat}\;\text{oxidation}\;\left(\text{mg}/\min\right)=1.6946\;{\text{VO}}_{2}\;\left(\text{mL}/\min\right)\;–\;1.7012\;{\text{VCO}}_{2}\;\left(\text{mL}/\min\right)$$

(2) $$\text{CHO}\;\text{oxidation}\;\left(\text{mg}/\min\right)=4.585\;{\text{VCO}}_{2}\;\left(\text{mL}/\min\right)\;–\;3.2255\;{\text{VO}}_{2}\;\left(\text{mL}/\min\right)$$

(3) $$\text{EE}\;\left(\text{kJ}/\min\right)=16.89\;{\text{VO}}_{2}\;\left(L/\min\right)+4.84\;{\text{VCO}}_{2}\;\left(L/\min\right),$$

where VO_2_ and VCO_2_ denote the measured rates of oxygen uptake and carbon dioxide production, respectively.

### 2.5 Exercise testing protocol

The exercise sessions for EE and substrate oxidation measurements were conducted on two occasions before and after the exercise training program. The first post‐training testing session was conducted within 1–2 days after the last training session. For each testing visit, the participants completed a treadmill exercise for 30 min at 50% HRR (pretraining) and 60 min at 60% HRR (post‐training). For the post‐training tests, instead of using the same intensity and duration as in the pretraining tests, we used the intensity and duration of the last training session. This approach allowed us to explore the accurate impact of ambient temperature on metabolic responses during postexercise recovery by simulating the conditions of a real‐life training session. The pretest and posttest exercise sessions were conducted in thermoneutral environments (22°C–23°C; 70% ± 5% humidity). The thermoneutral temperatures mirrored the ones used in fitness centers, which are generally selected to make exercising more comfortable and reduce the risk of heat illness compared with hot temperatures, particularly for obese individuals.

At the end of each exercise, a 5‐min active cool down was implemented before the participants were allowed to sit quietly in a climatic room for a 1‐h recovery period in either hot (31°C–32°C; 70% ± 5% humidity) or thermoneutral environments. The participants were subjected to either of the two conditions in a randomized crossover fashion, with 2–4 days of washout period separating the two tests. The metabolic measurements were performed during the recovery period. The cool down was performed using the same protocol in the thermoneutral environment for both the pre‐intervention and post‐intervention.

### 2.6 Body composition, body temperature, thermal sensation, and rating of perceived exertion (RPE)

The body composition of the participants was assessed before and after training using an eight‐electrode multifrequency bioelectrical impedance analyzer (Inbody 770, Korea). The measurements were scheduled in the morning after overnight fasting. To reduce potential dehydration, the participants were requested to drink 600 mL of water 2 h before arriving at the laboratory. A reminder was given to the participants on the day preceding each testing day, and a brief questionnaire was asked on the testing day to ensure that they complied with the hydration protocol. Urination was required within 15 min before the body composition measurement.

The participants' body temperature was continuously monitored throughout all exercise and recovery periods at the axillary area using an analog thermistor sensor probe (YSI 400 series, Yellow Springs Instrument Co., Inc.).

The participants rated their thermal sensation during recovery every 5 min using a scale of −4 (very cold) to 4 (very hot) (Tasing et al., 2016).

The participants' RPE was determined every 2 min during the exercise tests using the Borg 6–20 scale.

### 2.7 Statistical analysis

Data were analyzed using the Statistical Package for Social Science (SPSS), version 22.0 for Windows (IBM Corp.). Data are reported as mean ± standard deviation. A linear mixed model for the crossover trial was used to test for differences between substrate oxidation and EE during recovery in hot and thermoneutral environments before and after the training program. The paired *t*‐test was employed to test for pretraining versus post‐training differences in body compositions, substrate oxidation, and EE during rest. The paired *t*‐test was used to compare respiratory parameters during recovery between the two environments. Statistical significance was defined at *p* < 0.05 for all tests.

### 2.8 Ethical considerations

This study was conducted according to the ethical principles for human experimentation of the 1964 Declaration of Helsinki and with approval from the Institutional Review Board. All participants provided informed consent before participating in the study.

## 3 RESULTS

### 3.1 Characteristics of the participants

Among subjects who completed the study, the training included 12.8 ± 1.3 sessions on average (range, 12–15 sessions). The RPE of the participants was 12–13 (somewhat hard) during the pretraining exercise testing sessions and 14–15 (hard) during the post‐training exercise testing sessions. Although the body temperatures of the participants during postexercise recovery did not differ between the pretraining (thermoneutral, 35.5°C ± 0.9°C vs. hot, 36.0°C ± 0.9°C; *p* = 0.106) and post‐training (thermoneutral, 35.7°C ± 0.8°C vs. hot, 36.1°C ± 0.4°C; *p* = 0.058) testing sessions, the perceived temperatures (thermal sensation) during the recovery were higher in the hot than in the thermoneutral environment in both pretraining (thermoneutral, −1.3 ± 0.7 vs. hot, 1.9 ± 1.0; *p* < 0.001) and post‐training (thermoneutral, −1.2 ± 0.9 vs. hot, 1.9 ± 1.1; *p* < 0.001) tests.

### 3.2 Substrate oxidation and EE

#### 3.2.1 Tests during rest and exercise

There were no statistical pretraining versus post‐training differences in the resting levels of EE (pretraining, 63.7 ± 17.1 kcal/h vs. post‐training, 54.9 ± 12.3 kcal/h; *p* = 0.185) and fat (pretraining, 27.7 ± 17.4 mg/kg/h vs. post‐training, 23.6 ± 12.9 mg/kg/h; *p* = 0.367) and carbohydrate oxidation (pretraining, 61.4 ± 35.8 mg/kg/h vs. post‐training, 57.7 ± 43.1 mg/kg/h; *p* = 0.708). Furthermore, substrate oxidation and EE during exercise were similar between the hot and thermoneutral environments, both before and after the training period (*p* > 0.05). Pretraining versus post‐training comparison of substrate oxidation and EE during exercise was not performed because of the greater post‐training exercise intensity (50% HRR vs. 60% HRR) and duration (30 vs. 60 min) used for testing.

#### 3.2.2 Tests during postexercise recovery

**FIGURE 2:**
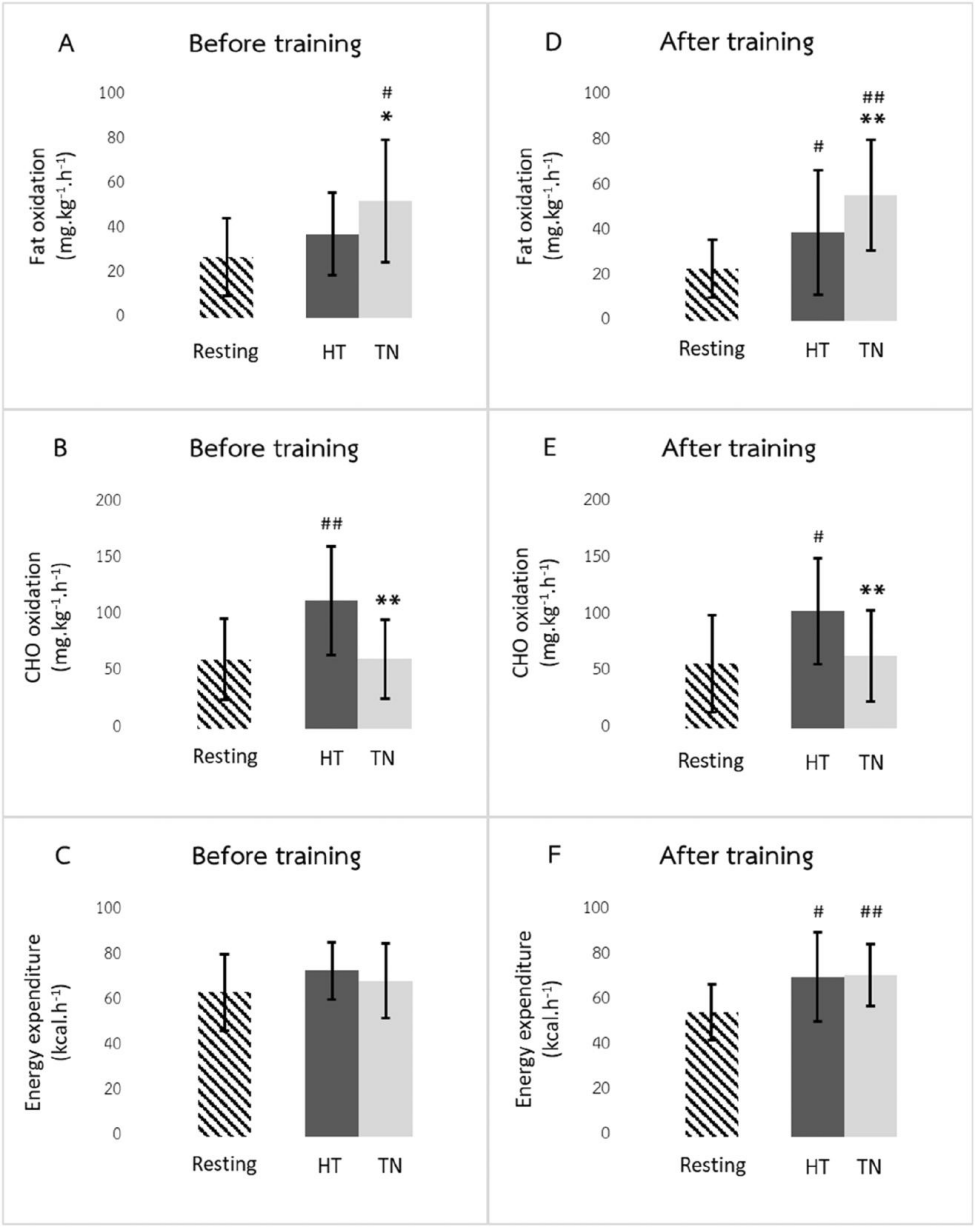
Substrate oxidation and energy expenditure during resting and post‐exercise recovery in hot (HT) and thermoneutral (TN) environments. Before training: (A) Fat oxidation, (B) carbohydrate (CHO) oxidation, and (C) Energy expenditure. After training: (D) Fat oxidation, (E) carbohydrate (CHO) oxidation, and (F) Energy expenditure (*N* = 16). Values are means ± SD: *Significant difference from recovery in hot environment (*p* < 0.01), **Significant difference from recovery in hot environment (*p* < 0.001), # Significant difference from the resting period (*p* < 0.01), ## Significant difference from the resting period (*p* < 0.001).

**FIGURE 3:**
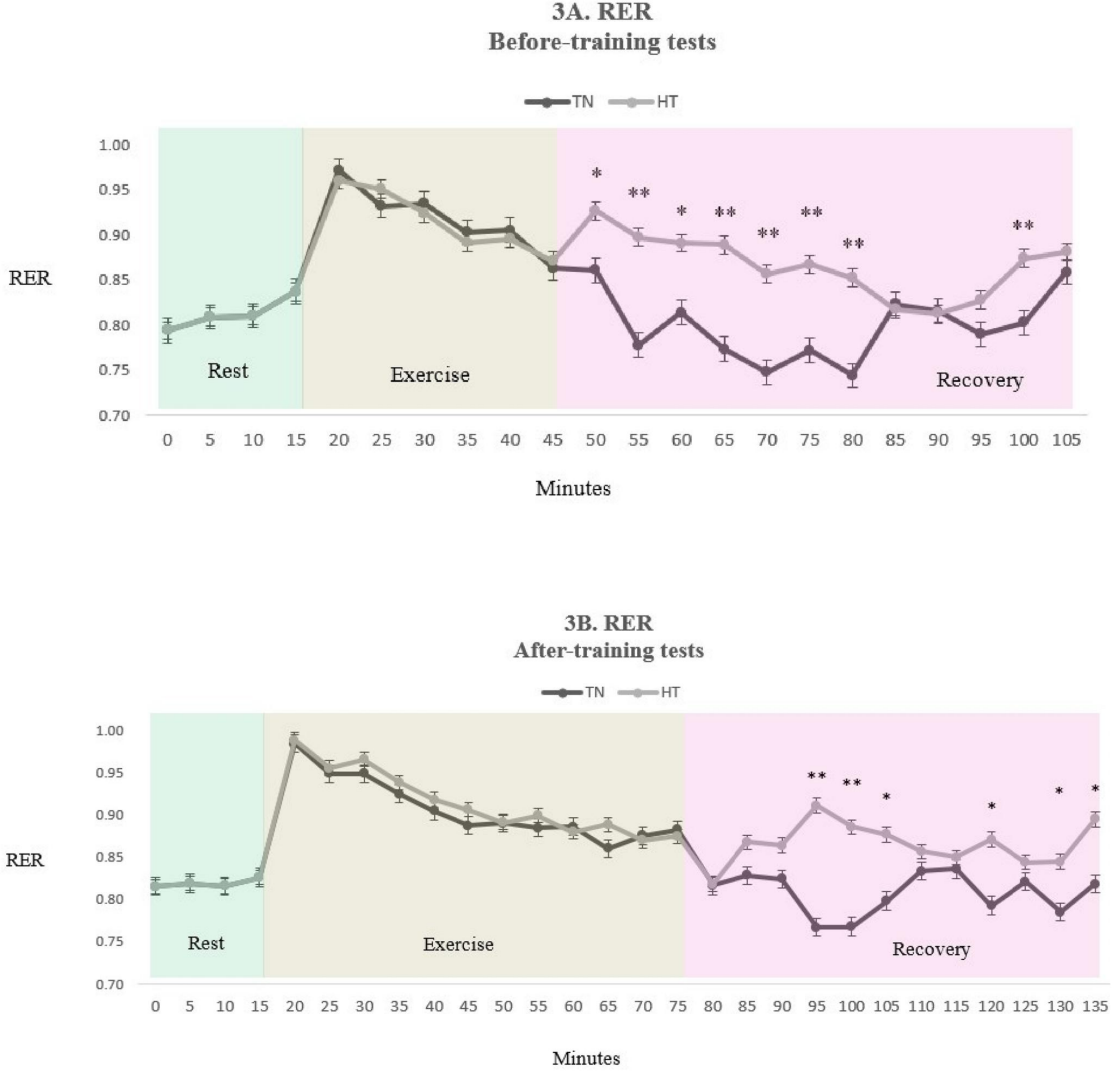
Time course of the respiratory exchange ratio during rest, exercise, and recovery (A) before training and (B) after training (*N* = 16). Values are means ± SD: *Significant difference between thermoneutral (TN) and hot (HT) environments (*p* < 0.05), **Significant difference between thermoneutral (TN) and hot (HT) environments (*p* < 0.001).

Before exercise training, fat oxidation during recovery was significantly higher in the thermoneutral (52.8 ± 27.4 mg/kg/h) than in the hot (38.0 ± 18.4 mg/kg/h; *p* = 0.01) environment (Figure 2A). Conversely, carbohydrate oxidation was lower in the thermoneutral (61.6 ± 35.4 mg/kg/h) than in the hot (113.4 ± 48.3 mg/kg/h; *p* < 0.001) environment (Figure 2B). The difference in EE during recovery in the two environments was not statistically significant (thermoneutral, 68.7 ± 16.5 kcal/h vs. hot, 73.3 ± 12.5 kcal/h; *p* = 0.314) (Figure 2C). The respiratory exchange ratio (RER) during recovery in the thermoneutral environment was lower than in the hot environment (Figure 3A).

The effects of environmental temperature on substrate oxidation and EE during recovery after exercise training were consistent with those before exercise training. Fat oxidation was significantly higher in the thermoneutral (56.0 ± 24.6 mg/kg/h) than in the hot (39.7 ± 27.5 mg/kg/h; *p* < 0.001) environment (Figure 2D). Furthermore, carbohydrate oxidation was lower in the thermoneutral (64.6 ± 40.5 mg/kg/h) than in the hot (104.0 ± 46.9 mg/kg/h; *p* < 0.001) environment (Figure 2E). Again, the difference in EE during recovery in the two environments was not statistically significant after training (thermoneutral, 71.3 ± 13.7 kcal/h vs. hot, 70.5 ± 19.6 kcal/h; *p* = 0.846) (Figure 2F). The RER during recovery in the thermoneutral environment was lower than in the hot environment (Figure 3B).

There were no carryover effects on substrate oxidation and EE between recovery in the hot and thermoneutral environments (*p* > 0.05 for all pretraining and post‐training periods). The comparisons of substrate oxidation and EE between rest and postexercise recovery before and after training periods are shown in Figure 2.

To determine whether hyperventilation occurred during recovery due to the hot environment, data on respiratory rate and ventilation were compared between the two recovery environments. The respiratory rate was lower in the thermoneutral than in the hot environment in both pretraining (thermoneutral, 17.6 ± 2.4 breaths/min vs. hot, 19.4 ± 3.9 breaths/min, and *p* = 0.008) and post‐training (thermoneutral, 19.1 ± 2.1 breaths/min vs. hot, 20.0 ± 2.4 breaths/min, and *p* = 0.023) tests. However, ventilation during postexercise recovery did not differ between the pretraining (thermoneutral, 7.49 ± 1.84 L/min vs. hot, 8.15 ± 2.01 L/min, and *p* = 0.178) and post‐training (thermoneutral, 7.60 ± 1.77 L/min vs. hot, 8.21 ± 1.67 L/min, and *p* = 0.182) testing sessions.

### 3.3 Body composition

The fat mass and body fat percentage significantly decreased with exercise training (*p* < 0.05). The other body composition indices did not change with exercise training (Table 1).

**TABLE 1:** Resting heart rate and body composition before and after exercise training program (*N* = 16).

	Before training	After training	Difference	95% CI	*p*‐value
Resting heart rate (bpm)	66.4 ± 10.6	62.7 ± 6.9	3.7 ± 10.3	−1.8–9.2	0.168
Body mass index (kg/m^2^)	33.3 ± 3.8	33.1 ± 3.7	0.2 ± 0.6	−0.1–0.5	0.125
Body mass (kg)	85.5 ± 13.4	84.9 ± 13.1	0.6 ± 1.4	−0.1–1.3	0.100
Muscle mass (kg)	25.9 ± 3.8	25.9 ± 3.7	0.0 ± 0.5	−0.2–0.3	0.878
Fat mass (kg)	38.6 ± 8.4	38.0 ± 8.6	0.6 ± 0.8	0.1–1.0	0.016*
Body fat (%)	44.8 ± 4.5	44.4 ± 4.9	0.4 ± 0.7	0.0–0.7	0.036*

*Notes*: Values are means ± SD. The “Before training” values were determined after 1 month of diet stabilization. Difference represents changes between before and after training. 95% CI represents a confidence interval of the difference.

*Significant difference between before and after training (*p* < 0.05).

## 4 DISCUSSION

We investigated the influence of environmental temperature on substrate oxidation and EE during postexercise recovery, both before and after short‐term exercise training, in overweight and obese women. Our main finding was that fat oxidation during postexercise recovery was greater in the thermoneutral than in the hot environment, both before and after exercise training. Furthermore, we found a lower RER during recovery in the thermoneutral environment, which served as independent evidence of the temperature dependence of substrate sources for metabolism. Finally, EE during postexercise recovery was unaffected by the ambient temperatures both before and after exercise training.

Excess postexercise oxygen consumption was observed in the recovery phase, which enhanced EE above the resting levels owing to more oxygen being required for lactate clearance, recovering the oxygen reserve, restoring the ATP/creatine phosphate, and maintaining thermoregulation and homeostasis (Borsheim & Bahr, 2003; Horton et al., 1998). EE was comparable between the two temperature conditions during recovery both before and after exercise training, suggesting that EE during the postexercise period was unaffected by environmental temperature or exercise training. In addition, we found that, relative to the resting condition, EE tended to elevate during postexercise recovery both before and after exercise training, showing a significant increase in the latter. The significantly higher EE during the 1‐h recovery period than the resting EE observed after training (Figure 2F) may be attributed to an ∼14% reduction in resting EE after training, although this magnitude of decrease was not significant compared with the pretraining levels. Whereas, during training, the participants progressed in their exercise capability to higher intensities and longer durations of exercise; EE during the postexercise period throughout training remained comparable with the pretraining stage. This suggests that the exercise stimulus before training (50% HRR, 30 min, and ∼150 kcal) and after training (60% HRR, 60 min, and ∼300 kcal) only induced slightly greater EE during the recovery as opposed to during rest. Although the training was progressive, the program was rather short (4 weeks). Given that the participants were sedentary, obese women, appropriate progression was practiced with the justification of the safety and benefits of the exercise program.

Although research has focused on substrate oxidation during rest and exercise in various environmental temperatures (Acosta et al., 2018; Gagnon et al., 2013; Haman et al., 2002; Jentjens et al., 2002; Maunder et al., 2020; Pilch et al., 2010; Tikuisis et al., 2000), little is known regarding the effect of temperature on fat and carbohydrate oxidation during postexercise recovery. Previous studies reported that postexercise EE and substrate oxidation were largely determined by exercise intensity and duration (Phelain et al., 1997; Warren et al., 2009). Because the exercise sessions preceding recovery in this study were independent but consistent with respect to intensity and duration for the two postexercise environments before and after training, the magnitude of EE during recovery did not differ. However, the fat and carbohydrate oxidation fractions were higher and lower, respectively, during recovery in thermoneutral environments compared to hot environments. The additional findings of lower and higher RERs in the thermoneutral and hot environments, respectively, confirm the pattern of selective, temperature‐dependent adjustment of energy utilization from fat or carbohydrate during recovery. These findings highlight the altered metabolic preferences for energy sources during postexercise recovery in the hot versus thermoneutral environments.

It should be noted that the RER is an indirect estimate of substrate use and that ambient heat‐induced hyperventilation may cause a higher RER (Tsuji et al., 2012). Therefore, we further analyzed the data on the respiratory rate and minute ventilation during postexercise recovery. It was found that the respiratory rate was slightly greater during recovery in the hot than in the thermoneutral environments for before (1.8 ± 2.4 breaths.min^−1^) and after training (0.9 ± 1.2 breaths.min^−1^). However, minute ventilation was not different between the two recovery environments for before and after training (all *p* > 0.05). These data indicate that a slight increase in the respiratory rate occurred due to the hot environment does not result in hyperventilation. Thus, an increase in the RER, which was interpreted as an increase in carbohydrate oxidation, was not caused by hyperventilation.

Previous cross‐sectional and prospective studies reported that postexercise EE, determined by excess postexercise oxygen consumption, decreased significantly after exercise at the same absolute intensity but remained unchanged after exercise at the same relative intensity in response to exercise training (Sedlock et al., 2010; Short & Sedlock, 1997). Similarly, another study reported that exercise training, which was part of the body mass management program, had no impact on EE and substrate oxidation during recovery after exercise at the same relative intensities, compared to before training (Lazzer et al., 2011). Unlike previous studies, our subjects performed the recovery trials after exercise at a higher relative intensity for a longer duration after, than before training. This inequality of exercise made training‐induced changes in postexercise EE and substrate oxidation unable to be determined directly. However, it is reasonably speculated that exercise training may have induced cardiovascular and metabolic adaptations to some extent, as seen by a trend to decrease resting heart rate and slight decreases in fat mass and % body fat. The effects of exercise training status, the preceding exercise, and environmental temperature would contribute together to recovery EE, and substrate oxidation revealed in our study.

The mechanism by which ambient temperatures differentially affect the body's preference for substrate oxidation during postexercise recovery remains unclear. A reciprocal control does exist between the proportion of fat and carbohydrate oxidation; when carbohydrate oxidation increases, fat oxidation decreases in a compensatory manner with a relatively small change in the total energy metabolism (Spriet, 2014). On this basis, one expects a higher carbohydrate oxidation in a hot environment at the expense of reducing fat oxidation. Carbohydrates likely remain the primary fuel source during increased metabolic demands, for example, for increased cardiac output and vasodilation to dissipate heat from the body in hot environments. The findings of this study support the notion that the body is metabolically flexible and has the capacity to rapidly alter metabolism in response to the demands of the body.

Previous studies reported that carbohydrate and fat metabolism could be improved even after a 2‐week moderate‐intensity exercise training in overweight and obese individuals (Lanzi et al., 2015; Malin et al., 2019). A metabolic change owing to short‐term exercise training was observed with changes in body composition markers (Lanzi et al., 2015) or without (Malin et al., 2019). In our study, only a slight and not significant tendency of reduced resting EE and body mass was detected. However, fat mass was observed to significantly decrease after a 1‐month progressive exercise training. This suggests that with the diet stabilization implemented in this study, there was a small training effect on body metabolism in the participants. Importantly, this training effect did not modify the influencing pattern of the environment temperatures on whole‐body preference for substrate oxidation during postexercise recovery, as evidenced by persistently higher fat oxidation during postexercise recovery in thermoneutral environments both before and after exercise training. In general, a good exercise program should be progressive, ensuring individuals eventually perform an exercise for longer durations and at higher intensities. Over time, the cumulative effects of repetitive exercise are reasonably expected to gradually modify EE and substrate oxidation in trained individuals. Thus, it would be interesting to know whether metabolic adaptation induced by long‐term exercise training would be different from what was found after our short‐term training.

### 4.1 Study strengths

To our knowledge, the present study is the first to compare substrate oxidation and EE during postexercise recovery between thermoneutral and hot environments in obese women. One of the main strengths of this study was the randomized crossover design and the controls for potential confounders. Because participants were females of reproductive age, we sought to minimize the effect of the menstrual period and internal and external hormonal influences on metabolism. To minimize food‐induced metabolic variation, participants consumed daily meals of the same amount and the dish type the day before each of the four testing days. Furthermore, we enforced diet stabilization for 1 month before and the additional month during exercise training to control the effect of changes in dietary intake and body mass induced by diets on metabolic adaptation.

### 4.2 Study limitations

The results of these experiments are based on short‐term, progressive exercise training. Thus, the results may change following a training program of greater intensity or longer duration to allow for more extensive metabolic adaptations. The exercise bouts immediately preceding the postexercise recovery period were employed at a lower intensity and shorter duration before (30 min and 50% HRR) than after (60 min and 60% HRR) the training program. These differences would confound a direct comparison of the effect of training on substrate oxidation and EE during postexercise recovery; the comparison requires a different experimental design. Further, it would be of interest to investigate the effect of environmental conditions on EE and substrate metabolism during postexercise recovery when the preceding exercise bout is more advanced according to the course of the exercise program. In addition, the participants in this study were all females of reproductive age, thereby limiting the generalizability of our findings. A future study of the same phenomena should include obese males and individuals of other ages. Fat oxidation and EE may increase for several hours during postexercise recovery; however, we only measured it for 1 h immediately after the exercise, and hence, the findings may differ with measurements taken beyond the first hour. Lastly, although the observed amount of EE and fat oxidation during recovery may seem modest, the long‐term health benefits from the cumulative effect of regular exercising in the right environmental temperature, recommended to obese individuals, should not be discounted and may have a measurable correlate during the recovery period.

## 5 SUMMARY AND CONCLUSION

Fat oxidation is higher following exercise in a thermoneutral than in a hot environment. However, postexercise EE was unaffected by environmental temperatures. This finding does not support the potential weight management benefit to obese women of selecting certain environmental temperatures during postexercise recovery to increase EE in the course of exercise training.

## CONFLICT OF INTEREST STATEMENT

The authors declare that there are no conflicts of interest regarding the publication of this paper.

## Data Availability

The data of this research study are available from the corresponding author and can be accessible upon reasonable request.
